# Prediction of Protein *S*-Nitrosylation Sites Based on Adapted Normal Distribution Bi-Profile Bayes and Chou’s Pseudo Amino Acid Composition

**DOI:** 10.3390/ijms150610410

**Published:** 2014-06-10

**Authors:** Cangzhi Jia, Xin Lin, Zhiping Wang

**Affiliations:** Department of Mathematics, Dalian Maritime University, Dalian 116026, China; E-Mail: xilinmath@163.com

**Keywords:** *S*-nitrosylation, post-translational modification, bi-profile Bayes, amino acid physicochemical properties

## Abstract

Protein *S*-nitrosylation is a reversible post-translational modification by covalent modification on the thiol group of cysteine residues by nitric oxide. Growing evidence shows that protein *S*-nitrosylation plays an important role in normal cellular function as well as in various pathophysiologic conditions. Because of the inherent chemical instability of the *S*-NO bond and the low abundance of endogenous *S*-nitrosylated proteins, the unambiguous identification of *S*-nitrosylation sites by commonly used proteomic approaches remains challenging. Therefore, computational prediction of *S*-nitrosylation sites has been considered as a powerful auxiliary tool. In this work, we mainly adopted an adapted normal distribution bi-profile Bayes (ANBPB) feature extraction model to characterize the distinction of position-specific amino acids in 784 *S*-nitrosylated and 1568 non-*S*-nitrosylated peptide sequences. We developed a support vector machine prediction model, iSNO-ANBPB, by incorporating ANBPB with the Chou’s pseudo amino acid composition. In jackknife cross-validation experiments, iSNO-ANBPB yielded an accuracy of 65.39% and a Matthew’s correlation coefficient (*MCC*) of 0.3014. When tested on an independent dataset, iSNO-ANBPB achieved an accuracy of 63.41% and a *MCC* of 0.2984, which are much higher than the values achieved by the existing predictors SNOSite, iSNO-PseAAC, the Li *et al*. algorithm, and iSNO-AAPair. On another training dataset, iSNO-ANBPB also outperformed GPS-SNO and iSNO-PseAAC in the 10-fold crossvalidation test.

## 1. Introduction

Protein *S*-nitrosylation, the covalent attachment of a nitric oxide (NO) moiety to cysteine residues of proteins resulting in the formation of *S*-nitrosothiols (SNO), is a typical redox-dependent posttranslational modification that is associated with redox-based cellular signaling [1,2,3]. Protein *S*-nitrosylation has been reported to play roles in the *in vitro*/*in vivo* regulation of a variety of metabolic enzymes, oxidoreductases, proteases, protein kinases, and protein phosphatases, as well as in the function of regulatory factors (including G protein) [4,5]. Many studies have shown that *S*-nitrosylated proteins exhibit abnormal increases or decreases in a variety of diseases [6]. For example, protein *S*-nitrosylation products were significantly increased compared with normal levels in diabetes, tuberculosis and other diseases; while protein *S*-nitrosylation products were significantly decreased compared with normal levels in asthma, neonatal oxygen deficiency, emphysema, and other diseases. Therefore, the regulation of protein *S*-nitrosylation modification may be a new and effective way for health protection. In addition, deregulation of *S*-nitrosylation has been implicated in tumor initiation and progression [4,7]. The increasing prominence of the roles of *S*-nitrosylation in diseases indicates a need for improved analytical methods to identify and quantify *S*-nitrosylated proteins under various physiological and pathophysiological conditions for investigative studies and clinical diagnosis [1,6,7]. The use of traditional mass spectrometry-based proteomics has been challenging because of the inherent chemical instability of the *S*-NO bond [4,8]. Currently, the biotin switch technique (BST), which was designed to purify and detect *S*-nitrosylated proteins, has become a widely used method for studying protein *S*-nitrosylation [9]. However, some researchers have suggested that the ascorbic acid signal enhancement as necessary and sufficient conditions of BST has led to a high number of false positives. A further study has shown that BST cannot be used to determine *S*-nitrosylated sites when the proportion of *S*-nitrosylated sites is less than 1% [10]. Hence, the computational prediction of protein *S*-nitrosylation sites may provide useful and experimentally testable information about potential protein *S*-nitrosylation sites. In recent years, several computational approaches have been developed to predict protein *S*-nitrosylated sites.

Hao *et al*. [11] developed the earliest prediction tool for *S*-nitrosylation called SNOSID, which is a support vector machine (SVM) system trained on the limited 65 *S*-nitrosylation sites and 65 non-*S*-nitrosylation sites that were available at the time. Xue *et al*. [12] constructed the first online server GPS-SNO for *S*-nitrosylation site prediction based on the modified group-based prediction system (GPS) version 3.0 algorithm. Trained on a large dataset of 504 experimentally verified *S*-nitrosylation sites in 327 unique proteins, GPS-SNO achieved an accuracy of 75.80%, a sensitivity of 53.57%, and a specificity of 80.14% in the jackknife cross-validation test. However, the independent predictive performance of GPS-SNO was tested on 485 *S*-nitrosylated substrates that were not identified by experimental verification; suggesting that further validation of the predictive capability of GPS-SNO is needed. In 2011, Lee *et al*. [13] and Li *et al*. [14] used different approaches to try to improve the prediction of protein *S*-nitrosylation. Lee *et al*. [13] incorporated information about amino acid composition, accessible surface area, and physicochemical properties into the maximal dependence decomposition (MDD) algorithm to obtain conserved *S*-nitrosylation motifs. Then, by combining the MDD-clustered motifs with a SVM, they built the online server SNOSite for predicting *S*-nitrosylation sites, which achieved an accuracy of 67.5% and a Matthew’s correlation coefficient (*MCC*) of 0.245. Li *et al*. [14] established the prediction model CPR-SNO, using a SVM to improve the prediction performance. Instead of a SVM, Li *et al*. [15] proposed a nearest neighbor algorithm model that incorporated maximum relevance minimum redundancy and incremental feature selection techniques; however, the prediction results were not very satisfactory. On a newly created training dataset and an independent testing dataset, the *MCC*s were only 0.1381 and 0.1886, respectively. Xu *et al*. [16] proposed a web server called iSNO-PseAAc, which incorporated position-specific amino acid propensity into pseudo amino acid composition. The iSNO-PseAAc predictor achieved a *MCC* of 0.3515, which is substantially higher than the best *MCC* of 0.1915 obtained by GPS-SNO. More recently, Xu *et al*. [17] developed a new predictor called iSNO-AAPair by taking into account the coupling effects for all the pairs formed by the nearest residues and the pairs formed by the next nearest residues along protein chains. Despite the many *S*-nitrosylation predictors that have been developed, the *MCC* prediction values that they achieve are relatively lower than the values achieved by predictors of other post-translational modifications. Therefore, the discovery of new features will help in the development of more effective tools for protein *S*-nitrosylation site identification.

Bi-profile feature extraction has been applied in the prediction of many types of protein post-translational modification and has provided significant improvements in prediction performance [18,19,20,21,22,23,24,25]. The theoretical basis of this approach is that positive and negative peptide sequences should exhibit different features or characteristics [18]. In this study, we propose a computational model iSNO-ANBPB based on an adapted normal distribution bi-profile Bayes (ANBPB) feature extraction model and Chou’s pseudo amino acid compositions for protein *S*-nitrosylation site prediction. We performed jackknife and 10-fold cross-validation experiments on two recently constructed training datasets in [15,16] and tested iSNO-ANBPB on an independent dataset constructed in [15], to comprehensively compare iSNO-ANBPB with four recently developed competing predictors. Three kinds of comparative results consistently indicated that iSNO-ANBPB achieved higher *MCC*s and outperformed other current approaches.

According to a recent comprehensive review [26] and demonstrated by a series of recent publications (see, e.g., [27,28,29,30]), to establish a really useful statistical predictor for a protein system, we need to consider the following procedures: (i) construct or select a valid benchmark dataset to train and test the predictor; (ii) formulate the protein samples with an effective mathematical expression that can truly reflect their intrinsic correlation with the target to be predicted; (iii) introduce or develop a powerful algorithm (or engine) to operate the prediction; (iv) properly perform cross-validation tests to objectively evaluate the anticipated accuracy of the predictor; (v) establish a user-friendly web-server for the predictor that is accessible to the public. Below, we describe how to deal with these steps.

## 2. Results and Discussion

### 2.1. Results

#### 2.1.1. Sequence Analysis of *S*-Nitrosylation Sites

To explore the distinction between *S*-nitrosylation peptide sequences and non-*S*-nitrosylation peptide sequences, we conducted sequence analysis on the Li training dataset [15]. We calculated the relative position-specific propensities of each amino acid at each position (*r_xj_*) in the sequence to obtain the relative frequency of a particular amino acid in the *S*-nitrosylation dataset over the frequency of the same amino acid in the non-*S*-nitrosylation dataset. As shown in Tables S1, several amino acids at specific positions revealed some distinctive *r_xj_* scores. Amino acids H, K, and N were found to be relatively enriched in the *S*-nitrosylation peptides with average *r_xj_* scores of 1.23, 1.25, and 1.13 respectively. On the other hand, amino acids C, F, and W were found to be relatively depleted in the *S*-nitrosylation peptides with average *r_xj_* scores of 0.64, 0.86, and 0.74 respectively. However, the independent distinct *r_xj_* scores are not sufficient for defining a sequence motif for *S*-nitrosylation sites and more complex patterns of position-specific residue propensities in peptide sequences should be exploited to further improve the computational performance of *S*-nitrosylation site predictors.

#### 2.1.2. Performance of the BPB, BRABSB, ANBPB and RANS Prediction Models

The weight parameters (*W*1 and *W*-1) in a SVM were adapted to increase the precision of sensitivity. For each training process, the initial *W*1 values were set to 1, 1.5, 2, and 2.5, until the *MCC*s reached their maximum. Notably, the performances of all these models significantly improved after the optimization of the *W*1 parameter (Tables S2–S5).

To find the best prediction model to identify potential protein *S*-nitrosylation sites, bi-profile Bayes (BPB) [18], bi-relative adapted binomial score Bayes (BRABSB) [23], adapted normal distribution bi-profile Bayes (ANBPB) [24], and the relative adapted normal score (RANS) [24] feature extraction combined with Chou’s pseudo amino acid composition were developed on the same Li training datasets. The performances of the BPB, BRABSB, ANBPB, and RANS models for predicting protein *S*-nitrosylation and non-*S*-nitrosylation sites were examined by jackknife tests. The weight parameter *W*1 was optimized separately for the BPB, BRABSB, ANBPB and RANS models and the detailed results are available in Tables S1–S4. The best results obtained by each model are listed in Table 1. The BPB and ANBPB models reached their highest *MCC* values of 0.2933 and 0.3014, respectively, for *W*1 = 2, while the BRABSB and RANS models reached their highest *MCC* values of 0.2949 and 0.2391, respectively, for *W*1 = 2.5. The ANBPB model achieved the best *MCC* value.

**Table 1:** Best predictive performances of four sequence encoding schemes.

Sequence Encoding Scheme	*W*1	*Sn* (%)	*Sp* (%)	*Acc* (%)	*MCC*
BPB + Ecomposition ^a^ + Scomposition ^b^	2	65.31	65.63	65.52	0.2933
BRABSB + Ecomposition + Scomposition	2.5	73.09	58.16	63.14	0.2949
ANBPB + Ecomposition + Scomposition	**2**	**67.60**	**64.29**	**65.39**	**0.3014**
RANS + Ecomposition + Scomposition	2.5	63.90	61.42	62.24	0.2391

^a^ Ecomposition denotes the composition of positively charged amino acids; ^b^ Scomposition denotes the composition of α-helix propensities of amino acids.

#### 2.1.3. Comparison of the Performance of iSNO-ANBPB with Current Computational Approaches

The classification performances of iSNO-ANBPB, the Li *et al*. method [15], SNOSite [13], iSNO-PseAAC [16], and iSNO-AAPair [17] were compared directly. Because there is no online server for the work done by Li *et al*. [15], iSNO-ANBPB and the Li *et al*. approach were both tested on the training dataset that was constructed in [15]. The results in Table 2 clearly show that iSNO-ANBPB outperformed the Li *et al*. method in the jackknife test. The *Acc* and *MCC* values achieved by iSNO-ANBPB are better by 3.78% and 0.163, respectively, than the *Acc* and *MCC* values achieved by the Li *et al*. method [15]. Further, using an independent Li test dataset, we tested the predictive power of iSNO-ANBPB to recognize novel *S*-nitrosylation sites and compared it with the power of the Li *et al*. method [15], iSNO-PseAAC [16], iSNO-AAPair [17], and SNOSite [13]. As shown in Table 2, the iSNO-ANBPB model achieved an overall accuracy of 63.41% and a *MCC* of 0.2984, which is better than the overall accuracies achieved by the other four methods. We also compared iSNO-ANBPB indirectly with the GPS-SNO predictor proposed by Xue *et al*. [12]. Xu *et al*. [16] reported that iSNO-PseAAC outperformed GPS-SNO when tested on the same benchmark dataset. Therefore, to make a fair comparison, we tested the performances of iSNO-ANBPB, GPS-SNO, and iSNO-PseAAC on the Xu training dataset. The iSNO-ANBPB model again achieved the best prediction performance, with an average accuracy of 70.77% and *MCC* of 0.4146, for the 50 times it was run in the 10-fold crossvalidation. The iSNO-PseAAC model achieved an average accuracy of 67.01% and a *MCC* of 0.3515, and GPS-SNO achieved the best average accuracy of 45.01% and *MCC* of 0.1915 with the threshold set at “low”.

**Figure 1:**
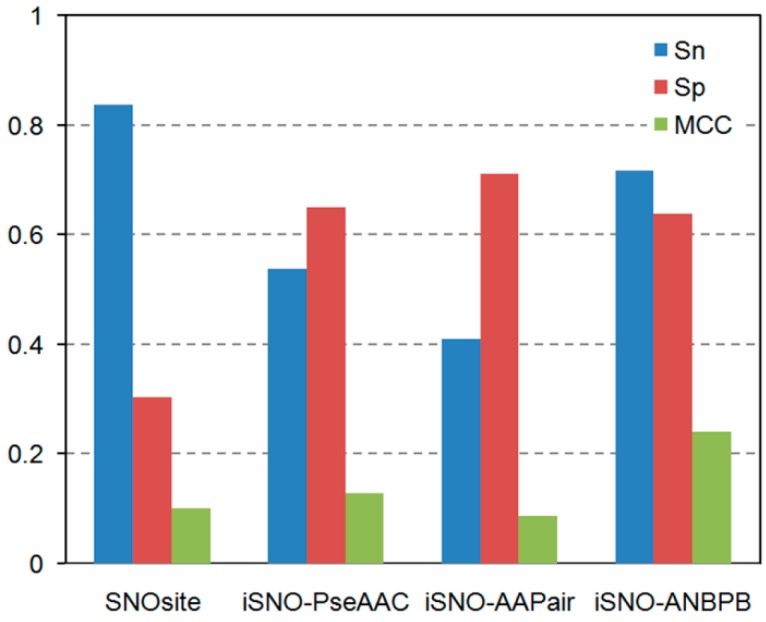
Potential *S*-nitrosylation sites predicted on 37 proteins through *S*-nitrosothiols (SNO)site, iSNO-PseAAC, iSNO-AAPair and iSNO-adapted normal distribution bi-profile Bayes (ANBPB) predictor.

To demonstrate the performance of our iSNO-ANBPB predictor, 37 experimentally-verified *S*-nitrosylated proteins which were not included in the training data set were studied. The sequences of such 37 proteins as well as *S*-nitrosylation site position are given in Supplementary Information. The detailed performances of SNOsite, iSNO-PseAAC, iSNO-AAPair, and iSNO-ANBPB against the 37 independent proteins are summarized in Figure 1. As can be seen from the table, iSNO-ANBPB outperformed the other three predictor in *MCC*, verifying the generalization ability of iSNO-ANBPB.

**Table 2:** Performance comparison of different computational approaches on different datasets.

Dataset	Methods	*Sn* (%)	*Sp* (%)	*Acc* (%)	*MCC*
Li training dataset	Li *et al*. [15]	42.86	70.98	61.61	0.1381
	iSNO-ANBPB	67.60	64.29	65.39	0.3014
Xu dataset	GPS-SNO ^a^	18.88	89.63	56.07	0.1210
	GPS-SNO ^b^	28.04	81.98	56.39	0.1193
	GPS-SNO ^c^	45.01	73.33	59.90	0.1915
	iSNO-PseAAC	67.01	68.15	67.62	0.3515
	iSNO-ANBPB	67.33	73.78	70.77	0.4146
Li test dataset	SNOSite	74.42	28.10	40.24	0.0248
	iSNO-AAPair	27.91	80.17	66.46	0.0858
	Li *et al*. [15]	51.16	69.42	64.63	0.1886
	iSNO-PseAAC	58.14	63.64	62.20	0.1940
	iSNO-ANBPB	74.12	59.50	63.41	0.2984

^a^ The data was derived from Table 1 in Xu *et al*. [16] and the threshold of GPS-SNO was set at “high”; ^b^ The data was derived from Table 1 in Xu *et al*. [16] and the threshold of GPS-SNO was set at “medium”; ^c^ The data was derived from Table 1 in Xu *et al*. [16] and the threshold of GPS-SNO was set at “low”.

### 2.2. Discussion

Protein *S*-nitrosylation plays a central role in regulatory mechanisms by fine-tuning protein activities associated with diverse cellular processes and biochemical pathway [1,3]. In addition, *S*-nitrosylation appears to have major roles in the etiology of a broad range of human diseases. However, the direct experimental identification of protein *S*-nitrosylation has been challenging, primarily because of the inherent chemical instability of the *S*-NO bond and low abundance of endogenous *S*-nitrosylated proteins [4,5]. Experimental identification of protein *S*-nitrosylation sites has other drawbacks such as expensive experimental costs, time-consuming experiments, and low specificity. Computational techniques have been developed to help overcome these drawbacks. Moreover, the recent experimental identification of hundreds of *S*-nitrosylation sites opens up the prospect of identifying *S*-nitrosylation sites by combining the experimental data with computer-based screening of peptide sequences.

In this study, we carefully examined the relative position specificity of each amino acid at each position, and identified distinctive amino acid enrichment/depletion profiles for peptide sequences in positive and negative datasets. To encapsulate these complex patterns of residue position-specific propensities for computational prediction, we constructed SVM prediction models using the ANBPB feature extraction approach combined with Chou’s PseAAC. ANBPB has been applied to predict protein *O*-GlaNAcylation sites and was shown to significantly improve prediction performance. The theory behind this approach is that the positive and negative profiles for encoding peptide sequences originate from an approximation of the binomial distribution, which can capture and exhibit the relative deviation of frequency of amino acids that surround the *O*-GlaNAcylation sites [24]. Apart from the ANBPB feature extraction, the physicochemical information of the amino acids in the peptide sequence was also considered because it has been demonstrated that the electrostatic charge of amino acids distantly located to a cysteine residue and amino acid propensities for secondary structure are critical for *S*-nitrosylation [15]. The resulting 42 features that we obtained were combined with the SVM classifier to construct our iSNO-ANBPB prediction model.

As described in the above sections, we also established BPB, BRABSB and RANS models to find the most appropriate predictor for protein *S*-nitrosylation. The theoretical distinctions among the four models have been discussed in [24] and the choice of models is determined by the sequence characteristics. For protein *S*-nitrosylation prediction, the ANBPB model gave the best performance, indicating that the ANBPB feature extraction approach may be more suitable than the BPB, BRABSB and RANS approaches for recognizing differences between *S*-nitrosylated and non-*S*-nitrosylated peptide sequences. We suspect that this finding may be because there is a degree of overrepresentation/depletion of certain features in *S*-nitrosylated and non-*S*-nitrosylated peptide sequences. The definition of BPB and BRABSB does not reflect enough the overrepresentation/depletion distinction, so they cannot detect *S*-nitrosylation sites as effectively as the ANBPB model.

We tested our iSNO-ANBPB model against GPS-SNO [12], SNOSite [13], the algorithm developed by Li *et al*. [15], iSNO-PseAAC [16], and iSNO-AAPair [17], because they are among the best *S*-nitrosylation prediction models that are currently available. We could not compare our iSNO-ANBPB model directly with the CPR-SNO predictor [14] because the web-server was not working. Using the Li training dataset, the iSNO-ANBPB model achieved an *Acc* of 65.39%, which is 3.78% higher than the *Acc* for the algorithm developed by Li *et al*. [15]. Using the Xu training dataset, the iSNO-ANBPB model achieved an *Acc* of 70.77%, which is 3.15% higher than the *Acc* achieved by the iSNO-PseAAC method and 11.27% higher than the best *Acc* achieved by GPS-SNO. Notably, the *Acc* achieved by iSNO-ANBPB using the Xu training dataset is about 5.38% higher than of the *Acc* using the Li training dataset, perhaps because the proportion of positive and negative samples in the Xu training dataset is close to 1. Using the Li test dataset, iSNO-ANBPB achieved a *MCC* of 0.2984, which is 0.1044 higher than the previous best-performing predictor iSNO-PseAAC [17], 0.1098 higher than method of Li *et al*. [15], 0.2126 higher than iSNO-AAPair, and 0.2736 higher than SNOSite. The results show that iSNO-ANBPB outperformed previous algorithms in term of precision, especially on independent testing datasets. These datasets are the most likely datasets to be selected for further experimental validation. Since user-friendly and publicly accessible web-servers represent the future direction for developing practically more useful models, simulated methods, or predictors [30,31], we shall make efforts in our future work to provide a web-server for the method presented in this paper.

## 3. Experimental Section

### 3.1. Datasets

To objectively and comprehensively compare our approach with other available approaches, we used two recently constructed datasets reported by Li *et al*. [14] and Xu *et al*. [15] (henceforth named the Li and Xu datasets, respectively). The Li training dataset contains 784 positive samples and 1568 negative samples from 499 proteins with <40% sequence similarity, while the Li test dataset contains 43 positive samples and 121 negative samples from 30 proteins with <40% sequence similarity. The Xu training dataset includes 731 positive samples and 810 negative samples from 438 proteins with <40% sequence similarity. Finally, we combined two of the training datasets and removed the redundant samples by by clustering program such as BLASTclust (http://toolkit.tuebingen.mpg.de/blastclust) [32]. The final 1229 positive samples and 1223 negative samples were used to construct the prediction model. After some preliminary trials and in the consideration of the previous works [14,15], we extracted 21-mer *S*-nitrosylation and non-*S*-nitrosylation peptides from both datasets for our analyses. If a possible *S*-nitrosylation site was located at the *N*- or *C*-terminus of the protein and the length of the peptide was less than 21 amino acids, the missing positions were filled with residue “X”s in this study.

### 3.2. Adapted Normal Distribution Bi-Profile Bayes Features Extraction (ANBPB)

Let *S= s_1_,s_2_,…,s_n_* denotes a peptide sequence, where s represents an amino acid, *i* (*i =* 1, 2, …, *n*) represents its position, and *n =* 21 represents the length of the peptide sequence in this study. According to bi-profile Bayes method [18], each of the training peptides can be encoded as (*p_1_,p_2_,…,p_n_,p_n+1_,…,p_2n_*), where (*p_1_,p_2_,…,p_n_*) represents the posterior probability of each amino acid at each position in the positive dataset and (*p_n+1_,p_n+2_,…,p_2n_*) represents the posterior probability of each amino acid at each position in the negative dataset. In this study, the frequency of each amino acid at each position was encoded as random variables *X_ij_*, *i* (*i =* 1, 2, …, 20) represents the *i*^th^ amino acid {A,C,D,E,F,G,H,I,K,L,M,N,P,Q,R,S,T,V,W,Y}, and *j =* 1, 2, …, 21 represents the *j*^th^ position. The random variables *X_ij_*, (*i =* 1, 2, …, 20; *j =* 1, 2, …, 21) are independent and obey the same binomial distribution *b(n*, *p)*, where *n =* 784/1568 is the number of peptide sequences in positive/negative set, *p* = 1/20 is the probability of each amino acid occurs in each position. Then the normal form variable 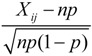 has a limiting cumulative distribution function which approximates a normal distribution *N*(0,1). Here, we modified the way of standard variable normalization to highlight and emphasize the distinction of each amino acid at the same position. We let *V_j_* denote the standard variance of X*_ij_* (*i =* 1, 2, …, 20), *i*.*e*., the deviation of frequencies of each at the same *j*^th^ position. And then we 
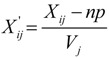
 as the new normalization of *X_ij_* and deemed it obeys the standard normal distribution. The posterior probability *p*_j_ (*j =* 1, 2, …, 2*n*) was coded by the adapted normal distribution as follows:

*p*_j_ = *P*(*X* ≤ *X_ij_*) = *ᵩ*(*X’_ij_*) (1)

where ᵩ(x) is the standard normal distribution function given by 
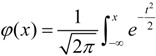
. For more details about this method, please refer to the original paper [24].

### 3.3. Pseudo Amino Acid Composition Based on Electrostatic Charge and Secondary Structure

To avoid losing many important information hidden in protein or peptide sequences, the pseudo amino acid composition [30,33] or Chou’s PseAAC [34] was proposed to replace the simple amino acid composition (AAC) for representing the sample of a protein or peptide. For a brief introduction about Chou’s PseAAC, and its recent development and applications, see a comprehensive review [26]. Since the concept of Chou’s PseAAC was proposed in 2001, it has rapidly penetrated into almost all the fields of computational proteomics, such as predicting protein submitochondrial localization [35], predicting protein structural class [36], identifying bacterial virulent proteins [37], predicting metalloproteinase family [38], predicting GABA(A) receptor proteins [39], predicting protein supersecondary structure [40], predicting cyclin proteins [41], classifying amino acids [42], identifying risk type of human papillomaviruses [43], identifying GPCRs and their types [44], predicting protein subcellular localization [45], and discriminating outer membrane proteins [46], among many others [26]. Because it has been widely and increasingly used, recently two powerful soft-wares, called “PseAAC-Builder” [47] and “propy” [48], were established recently for generating various special Chou’s pseudo-amino acid compositions, in addition to the web-server “PseAAC” (http://www.csbio.sjtu.edu.cn/bioinf/PseAAC/) [49] built in 2008.

As indicated by Lee *et al*. [13], Li *et al*. [15], and Marino *et al*. [50], the physicochemical properties of amino acids around cysteine residues can affect the occurrence of cysteine *S*-nitrosylation. Among these properties, electrostatic charge and propensity of secondary structure are critical for protein *S*-nitrosylation [15]. Accordingly, the 20 amino acids were divided into two different classes based on their electrostatic charge: positively charged amino acids (A): {A, C, D, E, H, L, P, Q, S, V, W} and negatively charged amino acids (G): {F, G, I, K, M, N, R, T, Y}. Similarly, based on their secondary structure, the 20 amino acids were divided into two other classes: α-helix propensities of amino acids (H): {C, D, G, N, P, S, T, W, Y} and other amino acids (E): {A, E, F, H, I, K, L, M, Q, R, V}. Owing to the summation of composition of pseudo amino acids (A) and composition of (G) is equal to 1, only one is independent. The same cases for the composition of pseudo amino acids (H) and composition of (E). So in practical calculations, the composition of positively charged amino acids (A) and α-helix propensities of amino acids (H) are adopted to construct the feature vectors.

### 3.4. Feature Space

According to the recent review [26], a peptide segment in our positive and negative datasets is formulated by

*P* = [*ψ*_1_,*ψ*_2_…,*ψ*_42_] (2)

where *ψ_i_*(*i* = 1,2,…,20) was defined by the posterior probability *p_i_* of each amino acid at each position in positive peptide sequences datasets; *ψ_i_*(*i* = 21,22,…,40) was defined based on the posterior probability *p_i_* of each amino acid at each position in negative peptide sequences datasets; ψ_41_,ψ_42_ were the composition of pseudo amino acids (A), and (H), respectively.

### 3.5. Support Vector Machine Implementation and Parameter Selection

An SVM is a set of related supervised learning methods used for classification and regression based on statistical learning theory. The SVM method has proven to be powerful in many fields of bioinformatics [18,19,20,51,52]. In this study, the SVM was trained with the LIBSVM package [53] to build the model and perform the predictions. The radial basis kernel function *k*(*x_i_,x_j_*) = exp{*−γǁx_i_−x_j_ǁ^2^*} was used for our SVM method. For different input features, the penalty parameter C and kernel parameter *γ* were optimized using the SVMcgForClass program [53] in the LIBSVM package based on a 15-fold cross-validation. The final parameters that we obtained were *C* = 22.6274 and *γ* = 0.03125. Optimized weight parameters (*W*1 and *W*-1) were set as 2 and 1 by looking for the best jackknife test results.

### 3.6. Performance Assessments

The jackknife test was used in this study to evaluate our method because it is considered as the most objective cross-validation method [31]. Sensitivity (*Sn*), specificity (*Sp*), accuracy (*Acc*) and *MCC* were used to quantify the performance of our method. They are defined as follows:

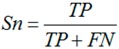
(3)

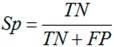
(4)

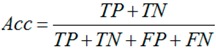
(5)

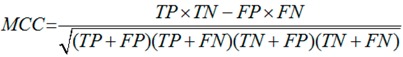
(6)
where *TP*, *TN*, *FP* and *FN* denote the number of true positives (correctly predicted *S*-nitrosylation sites), true negatives (correctly predicted non-*S*-nitrosylation sites), false positives (falsely predicted *S*-nitrosylation sites), and false negatives (falsely predicted non-*S*-nitrosylation sites), respectively.

## Supplementary Files

Supplementary File 1 Supplementary Information (PDF, 575 KB)
