# Supplementary material for: Prediction of Protein S-Nitrosylation Sites Based on Adapted Normal Distribution Bi-Profile Bayes and Chou’s Pseudo Amino Acid Composition
Source: Int J Mol Sci. 2014 Jun 10;15(6):10410–23. doi: 10.3390/ijms150610410 (PMC4100159; doi:10.3390/ijms150610410)
Supplement: Supplementary File 1 — Supplementary Information (PDF, 575 KB) [file ijms-15-10410-s001.pdf]

Supplementary Information

Table S1. The relative position-specific propensities of each amino acid at each position.

**A**	**C**	**D**	**E**	**F**	**G**	**H**
1.0608	0.6400	0.9923	0.9987	0.8570	1.0526	1.2277
**I**	**K**	**L**	**M**	**N**	**P**	**Q**
1.0897	1.2497	0.9639	0.9355	1.1295	0.9997	0.9530
**R**	**S**	**T**	**V**	**W**	**Y**	
1.1203	0.9166	1.0205	1.0007	0.7402	0.9247	

Table S2. Jackknife results for different weight parameters using BPB + Ecomposition + Scomposition.

W1	***Sn* (%)**	***Sp* (%)**	***Acc* (%)**	***MCC***
1	22.19	92.60	69.13	0.2121
1.5	50.51	77.30	68.37	0.2811
**2**	**65.31**	**65.63**	**65.52**	**0.2933**
2.5	74.23	54.91	61.35	0.2761

Table S3. Jackknife results for different weight parameters using BRABSB + Ecomposition + Scomposition.

W1	***Sn* (%)**	***Sp* (%)**	***Acc* (%)**	***MCC***
1	30.36	88.58	69.18	0.2338
1.5	52.42	74.36	67.05	0.2655
2	67.09	63.97	65.01	0.2936
**2.5**	**73.09**	**58.16**	**63.14**	**0.2949**
3	77.55	52.17	60.63	0.2836

Table S4. Jackknife results for different weight parameters using ANBPB + Ecomposition + Scomposition.

W1	***Sn* (%)**	***Sp* (%)**	***Acc* (%)**	***MCC***
1	30.87	88.33	69.18	0.2352
1.5	51.91	74.17	66.75	0.2586
**2**	**67.60**	**64.29**	**65.39**	**0.3014**
2.5	73.47	58.42	63.44	0.3009

Table S5. Jackknife results for different weight parameters using RANS + Ecomposition + Scomposition.

W1	***Sn* (%)**	***Sp* (%)**	***Acc* (%)**	*MCC*
1	34.82	85.52	68.62	0.2344
1.5	48.9	73.28	64.88	0.2128
2	58.55	66.45	63.82	0.2389
**2.5**	**63.90**	**61.42**	**62.24**	**0.2391**
3	65.82	56.57	59.65	0.2111

© 2014 by the authors; licensee MDPI, Basel, Switzerland. This article is an open access article distributed under the terms and conditions of the Creative Commons Attribution license (http://creativecommons.org/licenses/by/3.0/).
